# Sustainable Upgrade of Post-Consumer PLA: The Effect of Adding a Plasticizer and a Chain Extender on the Functional Properties and Toxicity of This Recycled Bioplastic

**DOI:** 10.1021/acsomega.5c09603

**Published:** 2025-12-22

**Authors:** Diana Morán, Eliezer Velásquez, Marta Arroyo Calatayud, Beatriz de la Fuente, Pilar Hernández-Muñoz, Carol López-de-Dicastillo

**Affiliations:** † Packaging Group, 83071 Institute of Agrochemistry and Food Technology (IATA-CSIC), Av. Agustín Escardino 7, Paterna 46980, Spain; ‡ Technology Center for Packaging Innovation (LABEN-Chile), 28065 University of Santiago of Chile (USACH), Obispo Manuel Umaña 050, Santiago 9170201, Chile; § Center for the Development of Nanoscience and Nanotechnology (CEDENNA), University of Santiago of Chile (USACH), Av. Libertador Bernardo O’Higgins 3363, Santiago 9170124, Chile; ∥ Research Group in Integrative Approaches in Food Toxicology, Institute of Agrochemistry and Food Technology (IATA-CSIC), Av. Agustín Escardino 7, Paterna 46980, Spain

## Abstract

The environmental risks associated with the use of petroleum-based plastics are well known, and therefore, the use of biopolymers has boomed in recent years. Polylactic acid (PLA) stands out as a great alternative, and although PLA is compostable, its recycling is considered a sustainable approach to manage its residues and promote a circular economy. The aim of this work was to investigate the final properties of post-consumer recycled PLA (rPLA) after the addition of different additives. Post-consumer PLA-based plastics were obtained after a simulation of service life and recycling processes of commercial PLA-based water bottles, and subsequently, different composites including a plasticizer and a chain extender (CE) were obtained by melt extrusion. Significant changes were observed in the structural and thermal parameters of the composites, including their crystallinity, when these additives were incorporated. However, the water vapor permeability values were not significantly different from that of control rPLA. In terms of overall migration, the values obtained for samples containing CE were well below the established limit, although samples containing plasticizers showed quite high values. Cytotoxicity assays in intestinal cells indicated that only composites with the highest plasticizer content exhibited slight toxicity at the intestinal barrier. Overall, contrasting roles were identified for the two additives: the plasticizer enhanced the flexibility but led to increased migration values, whereas the CE improved stability and reduced the migration risk. These results demonstrated that modifying post-consumer rPLA with suitable additives can improve its physical properties while maintaining safety, thus offering a practical and sustainable approach to its use in food packaging applications.

## Graphical abstract

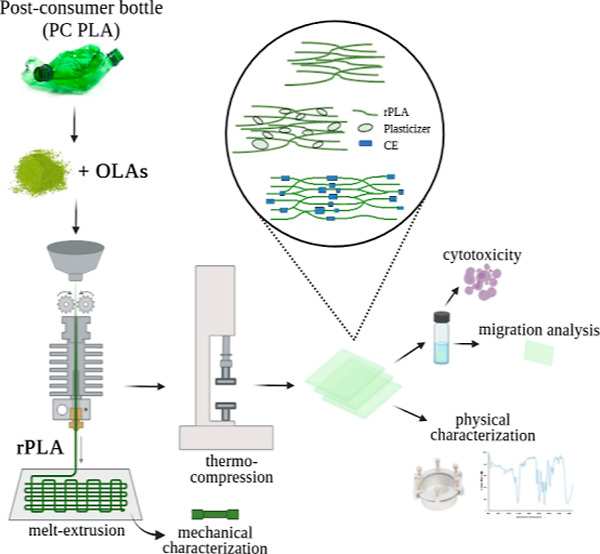

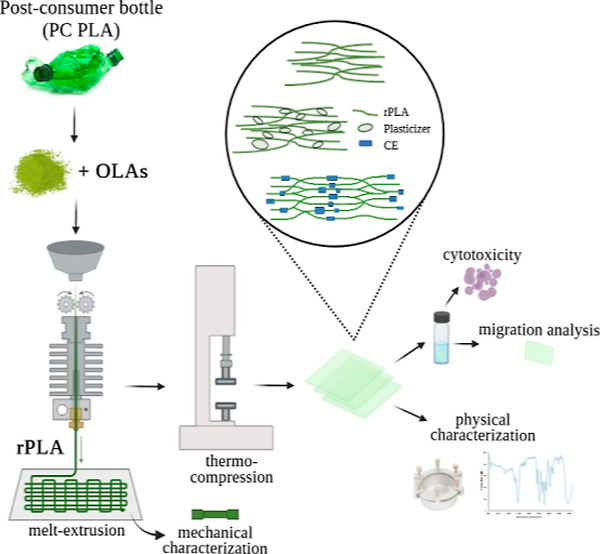

## Introduction

It is well known that petroleum-based plastics are the most widely used materials in many industries due to their great properties and abundance and the low cost of petroleum as a raw material, which in turn meet the performance requirements of many applications. However, their use contributes significantly to a number of ecological and environmental problems,^1^ as they take hundreds of years to degrade and also contain various additives and other toxic compounds. This has led to the search for alternatives to replace conventional polymers, and there has been considerable research into the use of polymer synthesis using renewable resources for the development of biopolymers.^2^ Biopolymers refer to a range of plastics that are really attractive due to their biocompatibility and biodegradability as they are obtained from renewable biomass sources, such as sugar, starch, or vegetable oils.^3^

Polylactic acid (PLA) is considered a promising alternative to petroleum-based polymers and a potential green circular material with a low environmental impact.^4^ PLA is a linear aliphatic thermoplastic polyester derived from lactic acid, which exists in two stereoisomeric forms, l-lactic acid and d-lactic acid. The polymerization of these isomers leads to the formation of PLA. Depending on the ratio and distribution of l- and d-lactic acid units within the polymer chains, various forms of PLA can be synthesized, such as poly­(l-lactic acid) (PLLA), poly­(d-lactic acid) (PDLA), or racemic products,^5^ yielding PLA materials with diverse mechanical and thermal properties. PLA can be processed into a wide range of products using various thermal processing techniques, such as extrusion, injection molding, blow molding, and thermoforming.^1,6^ At the same time, it is widely used in many sectors, such as the pharmaceutical,^7^ cosmetics,^8^ or food packaging industries,^9^ among others.

Although PLA has many advantages in terms of structural and physical properties, such as good transparency, mechanical strength, and ease of processing, it also has certain limitations. Its high glass transition temperature (*T*_g_ ∼60 °C) makes it very fragile, and its low elongation at break and high stiffness make it susceptible to fracture under mechanical stress.^10^ These characteristics limit its use in flexible applications, for instance packaging films.^11^ To mitigate these limitations, the development of polymer composites based on the combination of PLA and additives has been widely used in recent years. On the one hand, plasticizers, which can be low-molecular-weight polymers, oligomers, or organic compounds, are known to efficiently improve flexibility, processability, and impact resistance of rigid polymers by lowering their *T*_g_.^12^ Thus, biobased plasticizers, derived from renewable sources, such as vegetable oils, starch, cellulose, citric acid, or lactic acid, represent a particularly attractive option as they are a group of environmentally sustainable plasticizers.^12^ On the other hand, another limitation of PLA is its susceptibility to thermal degradation, thermo-oxidative, thermo-mechanical, and hydrolytic degradation during processing.^13^ This can be avoided by increasing its molecular weight by adding chain extenders (CEs) and additives that^14^ act as coupling agents, promoting intermolecular interactions and strengthening the structure of the polymer, which in turn improve its mechanical properties.^15^ Previous works have reported that plasticizers commonly used to improve the flexibility of PLA are typically employed at concentrations between 15 and 30 wt % with respect to the polymer content, and they are responsible for decreasing *T*_g_ and increasing chain mobility while maintaining the processability of the polymer. According to this, Alijanian et al. (2024) developed glycerol-based oligomeric resins functionalized with oligo­(lactic acid) arms to modify PLA, mimicking its chemical structure. They investigated the effect of arm length (three vs seven lactic acid units) and concentration (10–20 wt %) on PLA films. The most pronounced improvements were obtained using the shorter-chain modifier at the highest concentration.^16^ Soybean oil derivatives are also widely used as plasticizers for PLA. Recently, Yaman et al. (2024) formulated antimicrobial PLA films using a multicomponent system containing epoxidized soybean oil (20 wt %) and spruce resin (15 wt %) as plasticizers, which together enhanced the physical, thermal, and water vapor barrier properties of the films.^17^ Conversely, CEs are typically added at significantly lower concentrations (1–5 wt %) due to their high reactivity with PLA terminal groups. Previous studies on polyesters modified with CEs have demonstrated the efficacy of these additives in promoting extension and ramification reactions. Tang et al. (2021) reported that the molecular weight of polybutylene adipate terephthalate (PBAT) increased when modified with Joncryl ADR 4370, a commercial CE, at very low content (0.5–1.5 wt %).^18^ Because PBAT and PLA are both biopolyesters, the reaction mechanism between the CE and terminal groups is analogous, making the comparison relevant at the reaction level. In a recent study, Luo et al. (2024) investigated the use of epoxidized cardanol oleate as a biobased CE for PLA. Its incorporation between 1 and 2 wt % increased the molecular weight, improved the thermal stability, and increased the melt viscosity of PLA, resulting in an improved performance during processing.^14^

Despite the biodegradability of PLA through industrial composting, a sustainable approach is needed for proper waste management and environmental protection within a circular economy model. In this respect, when reuse is no longer an option, recycling is prioritized as a key factor for this purpose. Specifically for PLA, recycling not only extends the life of the polymeric materials but also contributes economically to the biopolymers market by ensuring its circularity. In addition, there are three key reasons for the compostability of PLA that underline the need for recycling: (1) large amounts of discarded PLA could cause environmental problems in the future due to high methane emissions; (2) direct compostability of PLA packaging means a loss of valuable raw materials, so recycling is essential to reduce the consumption of renewable resources needed to produce the corresponding monomers; and (3) the supply of post-consumer PLA exceeds the current demand for compost. It has been discussed that recycled PLA can be a good alternative to virgin PLA if its properties are properly controlled and modified.^19^ This can be achieved by melt-blending with other polymers or even by adding various additives, such as plasticizers, antioxidants, or CEs. In addition to an environmental protection approach to recycling and reuse, food contact materials must be evaluated from a food safety perspective. Migration testing is essential in the risk assessment of food packaging, especially if recycled plastics are incorporated. Furthermore, information about the potential toxic effects of migrating substances is required for a comprehensive evaluation of human health risk. Although the use of additives such as plasticizers and CEs in virgin PLA has been extensively studied, there is very little literature on their application in recycled PLA, highlighting the exploratory and novel nature of this study. In addition, the starting material of this study was a post-consumer PLA-based water bottle, whose composition includes the PLA polymer and additives that can affect and possibly hinder their effect.

In this context, the sustainable approach referred to in this work proposes a strategy for recovering post-consumer recycled PLA that combines mechanical recycling with the use of compatible additives based on renewable resources. This strategy extends the useful life of PLA without the use of virgin raw materials or petrochemical modifiers, thereby reinforcing the circular economy model applied to bioplastics. Therefore, in this work, commercial acid lactic oligomers (OLAs)-based additives have been incorporated within post-consumer PLA-based water bottles in order to investigate their effect on the technological and safety properties of this recycled plastic. First, post-consumer recycled PLA was obtained through a simulated useful life and recycling processes of commercial PLA-based water bottles involving accelerated bottle aging and common washing and drying steps, allowing for a more realistic simulation of a secondary mechanical recycling process.^20^ Subsequently, different composites were developed through melt extrusion by incorporating a plasticizer and a CE at different concentrations, and a thorough characterization of their structural, thermal, mechanical, and barrier properties was carried out. Plasticizer concentrations of 20 and 30 wt % and CE concentrations of 3 and 5 wt % were selected to evaluate their effect on rPLA within the functional ranges typically reported in the literature, while ensuring that its processability and stability were not compromised. Finally, a preliminary study of global migration of developed composites and their cytotoxicity studies were also assessed.

## Experimental Section

### Materials

Commercial green PLA-based water bottles were supplied by the Cabreiroá company (Hijos de Rivera S.A.). Sodium hydroxide (NaOH) was purchased from Fluka Biochemika (Barcelona, Spain) and the TritonTM X-100 surfactant was supplied by Sigma-Aldrich (Madrid, Spain). Silica gel 2.5–6 mm with an indicator (without cobalt chloride) was purchased from PanReac (Barcelona, Spain). Glyplast OLA 2 (plasticizer) and Glyplast OLA 550 (CE) were purchased from Condensia (Barcelona, Spain). Ultrapure water was obtained from a Milli-Q Plus purification system (Millipore, Molsheim, France). PLA-based bottles contain 50 cL of water. Their weight was 27 g and thickness was around 450 μm in the bottle body, increasing to 700 μm in the top of the bottle shoulder (a photograph of this bottle can be found in Supporting Information, Figure 1SM1).

**Figure 1:**
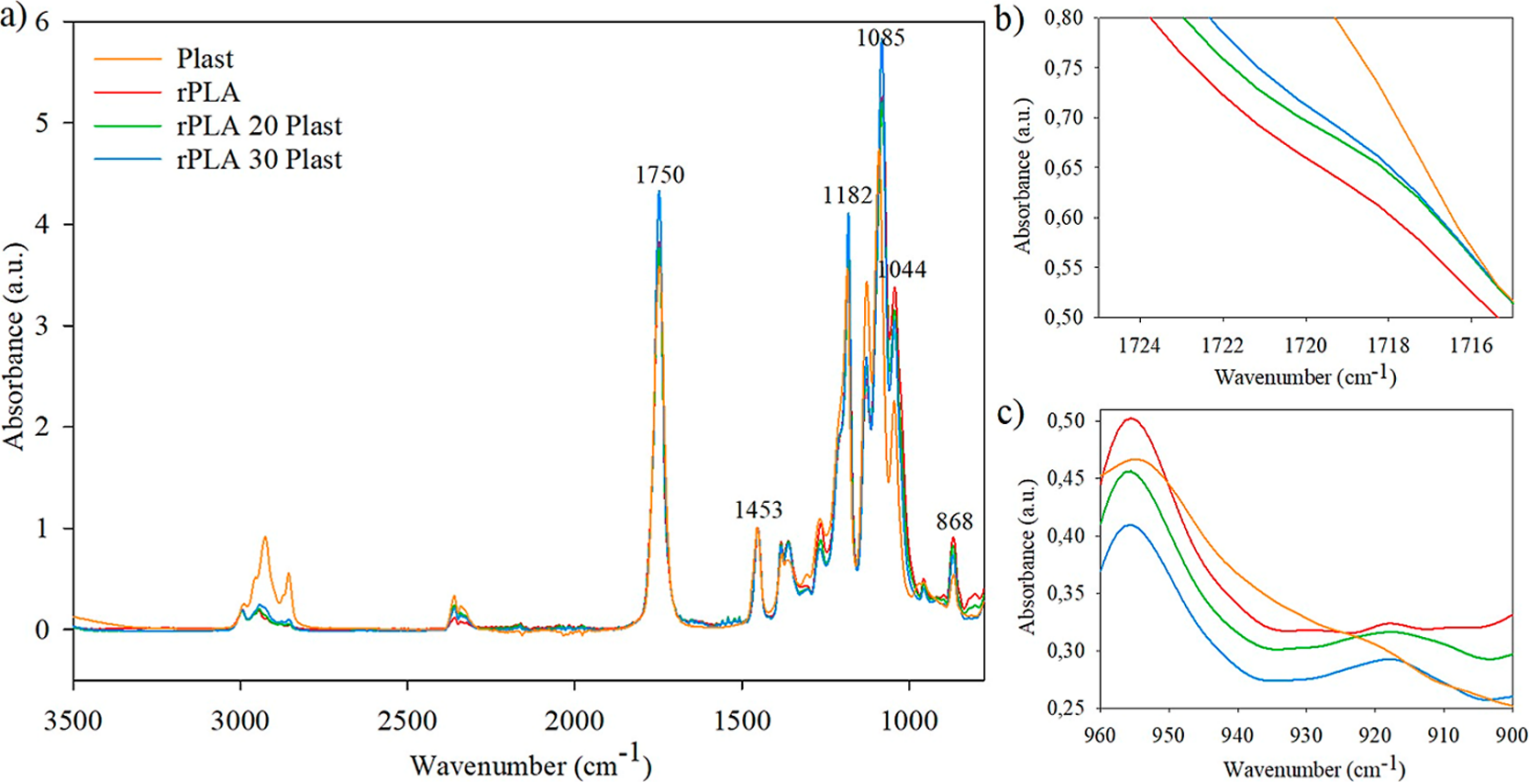
Comparison of the FTIR spectra of the plasticizer, rPLA, and films with different plasticizer concentrations between: (a) 850 and 3500 cm^–1^; (b) 1715 and 1725 cm^–1^; and (c) 900 and 960 cm^–1^.

### Secondary Mechanical Recycling Simulation

PLA-based water bottles were subjected to a sequential accelerated aging and recycling simulation to obtain the “post-consumer” recycled PLA plastic (PC-PLA).^21,22^ Basically, the bottles were subjected to a photochemical degradation step, where they were exposed to UVB radiation for 40 h, followed by a thermal degradation step, where they remained in a convection oven at 50 °C for 468 h, and finally a hydrothermal degradation step, where they were placed in a distilled water bath at 25 °C for 240 h. This was followed by a washing step with a 1 wt % NaOH solution and a 0.3 wt % Triton-X surfactant solution at 85 °C for 15 min. The bottles were rinsed with distilled water and dried at 40 °C for 72 h to obtain the post-consumer recycled bottle named PC-PLA.

### Development of Composites of rPLA with OLA-Derived Additives

The post-consumer bottles were mechanically crushed, and prior to reprocessing the composites by melt extrusion, additives were added at different concentrations to PC-PLA and manually mixed. Commercial Glyplast OLA 2 plasticizer (Plast) was added at 20 and 30 wt % with respect to polymer weight (samples rPLA 20 Plast and rPLA 30 Plast, respectively); and commercial Glyplast OLA 550 CE was added at 2 and 5 wt % with respect to rPLA weight (samples rPLA 2 CE and rPLA 5 CE, respectively). The samples were processed through melt extrusion in a corotating twin-screw extruder MC 15 HT (L/D = 30) (Xplore, Barcelona, Spain) at 180 °C and a rotation speed of 100 rpm for a residence time of 3 min, obtaining the samples as filaments. These filaments were ground and converted into films by thermo-compression using a Micro Scientific LP20-B hot plate (Labtech engineering Samut Prakan, Thailand) at 160 °C, 70 bar for 3 min, followed by 0.5 min cooling. These films were used for further characterization of structural, thermal, and barrier properties, as well as global migration tests. Injection molded specimens were also processed in order to analyze the mechanical properties of the developed composites according to ASTM D638 Type IV (2 mm thick and dimensions of 75 mm × 10 mm); dog bone-type specimens were obtained using an IM 12 (Xplore, Sittard, The Netherlands). Piston and mold temperatures were set at 180 and 40 °C, respectively, and the injection was carried out at 4 bar for 1 s. The material was held at 5 bar for 4 s, and the cooling time was 10 s. Samples without plasticizers and CEs were also prepared in the same way for comparison with those containing additives and were named rPLA.

### Structural Characterization

#### Fourier Transform Infrared (FTIR) Analysis

FTIR spectroscopy using a JASCO 4100 FTIR spectrometer (Jason, Easton, MD) with a single reflection attenuated total reflectance accessory (ZnSe crystal, PIKE Technologies, USA) was used to observe possible structural changes in PLA. Spectra were measured between 700 and 4000 cm^–1^ with 64 scans and a resolution of 4 cm^–1^. The FTIR-ATR spectra were corrected using Spectra Manager version 2.15.01 and normalized to the band at 1453 cm^–1^, which has been reported as the internal standard of PLA.^23^

#### Molecular Weight

The number-average molar mass ( ${\mathrm{M̅}}_{n}$ ) and weight-average molar mass ( ${\mathrm{M̅}}_{w}$ ) of the samples were measured by gel permeation chromatography (GPC), using a Waters Separation Module-Alliance 2695 (Waters, Barcelona, Spain), coupled to a diode array ultraviolet (UV) detector PDA 996 set to 220 nm, and Waters Styragel HR 5E, 4E, and 3 HPLC columns, combined in series. The mobile phase used was the THF isocratic regime, with a flow rate of 0.3 mL/min at 35 °C and an injection volume of 10 μL. The molecular weight distribution was calculated relative to certified mass polystyrene standards (580–3.080.000 Da).

### Thermal Characterization

#### Thermogravimetric Analysis

Thermogravimetric studies were performed on a TGA 550 thermobalance (TA Instruments, New Castle, USA). 10 mg of each sample was placed in 70 μL of standard alumina vessels and heated from 30 to 800 °C at 10 °C min^–1^ under a nitrogen atmosphere. The temperature of maximum degradation was registered. The analysis was carried out in duplicate for each sample.

#### Differential Scanning Calorimetry

Differential scanning calorimetry (DSC) analyses were carried out by using a DSC Q2000 instrument (TA Instruments, New Castle, USA). 10 mg of samples was placed into aluminum capsules and subjected to three thermal processes: (i) −20 to 220 °C (first heating); (ii) 220 °C to −20 °C (cooling); and (iii) −20 to 220 °C (second heating), with a heating/cooling rate at 10 °C min^–1^ under a nitrogen atmosphere. Glass transition temperature (*T*_g_), maximum melting temperature (*T*_m_), cold crystallization temperature (*T*_cc_), and their corresponding enthalpies, Δ*H*_m_ and Δ*H*_cc_, were determined using TA Instruments Universal Analysis 2000 software by analyzing the thermograms during the second heating process. The crystallinity (χ_c_) of PLA samples was calculated using eq 1:

1 $$\chi_{c}\left(\%\right)=\frac{{\Delta H}_{m}-{\Delta H}_{cc}}{{\Delta H}_{\infty }}\times 100$$

with the theoretical melting enthalpy for a fully crystalline PLA (Δ*H*_∞_) being 93.1 J g^–1^.^21^ DSC analysis was carried out in duplicate.

### Barrier and Mechanical Properties

#### Water Vapor Permeability

WVP tests were carried out at 50 and 90% RH and RT using Payne permeability cups (Elcometer, Manchester, UK) following ISO 2528.^24^ The cups were filled with 4 g of silica gel and sealed with vacuum silicone (Sigma, Barcelona, Spain). The films were also fixed with a flat aluminum ring and three pressure screws, leaving a permeable surface of 4.91 cm^2^. The cups of each sample were stored in desiccators containing salt solutions of magnesium nitrate and potassium nitrate at 50 and 90% RH, respectively. The cups were weighed daily for 6 days to obtain the weight gain versus time graph that provided the slopes to calculate the WVP values, considering the water pressure gradient, the thickness of each film, and the area used to calculate the WVP.

#### Mechanical Properties

Tensile testing of the specimens was performed using a 34TM-5 Universal testing machine (Instron, Barcelona, Spain) equipped with a 1 kN load cell, and the parts were stretched at 60 mm min^–1^ until failure. Young’s modulus (YM), tensile strength (TS), and elongation at break (εb) were calculated from stress–strain curves according to ASTM D638. For each material, eight measurements were performed.

### Global Migration Analysis

Global migration studies of developed composites were carried out in two food simulants, Sim A (10% ethanol), assigned to aqueous foods, and Sim D1 (50% ethanol), assigned to fatty foods (Commission Regulation (EU) 2016/1416). Double-sided total immersion migration tests were performed by immersing a rectangular strip of the films (1 cm × 3 cm) in 5 mL of simulants in glass-stoppered tubes with PTFE closures. Migration tests were performed at 10 and 40 °C for 10 days of storage. The total amount of compounds transferred was determined by the difference in weight before and after the contact days after the films had been dried in a vacuum oven at 40 °C for 2 days.

Tests were also performed in distilled water under the same conditions for subsequent cytotoxicity tests since the presence of ethanol in the simulants would negatively affect cell viability. According to Regulation (EU) No. 10/2011, water is also considered a food simulant in overall migration assays.

### Cytotoxicity Assay in Intestinal Cells

#### Cell Culture Line and Maintenance

The human colon carcinoma Caco-2 cell line was acquired from the American Type Culture Collection (ATCC HTB-37). Enterocyte cells were maintained in Dulbecco’s Modified Eagle’s Medium–high glucose, including l-glutamine, sodium pyruvate, and sodium bicarbonate, and supplemented with 10% (v/v) fetal bovine serum, 100 U/mL of penicillin, 0.1 mg/mL of streptomycin, and 0.0025 mg/L of amphotericin B (DMEMc). All reagents were obtained from VWR and Hyclone. Cells were cultured in 75 cm^2^ flasks and maintained at 37 °C with an air atmosphere containing 5% CO_2_ and 95% relative humidity. The medium was changed every 2–3 days, and the cells were detached using a solution of trypsin (0.5 mg/mL) and EDTA (ethylenediaminetetraacetic acid, 0.2 mg/mL) once the cell monolayer reached 80–90% confluence. The in vitro assays were performed using Caco-2 cells between passages 11 and 15.

#### Exposure Treatments

The aqueous solutions obtained in the global migration tests of control rPLA and additivated rPLA composites in water were used to evaluate the potential intestinal toxicity. Since cell exposure treatments must have a physiological pH and osmolarity, aqueous solutions cannot be applied directly to intestinal monolayers. To overcome this drawback, powdered culture medium (Gibco) was reconstituted in water (control treatment) and the corresponding aqueous migration solutions (exposure treatments under study). They were then supplemented in the same manner as for the liquid medium used for cell maintenance. Neutral pH values and osmolarity values in the range of 280–320 osm were confirmed for all treatments before being applied on Caco-2 cells.

#### Cell Viability Assay

The in vitro assay was conducted using 96-well plates, with a seeding density of 1 × 10^4^ cells/cm^2^. Following 7 days of cell growth, the intestinal cell monolayers were exposed to control and aqueous migration solutions for 24 h. After exposure, cell viability was determined using the resazurin (7-hydroxy-3H-phenoxazin-3-one-10-oxide sodium salt) (Sigma, Spain) method. Briefly, 30 μL of resazurin solution (100 μg/mL in DMEMc) was added to the wells, and the plate was then incubated (37 °C, 5% CO_2_, and 95% relative humidity) for approximately 2 h. A color difference between treatments and control was observed. As viable cells can convert blue resazurin to pink resazurin via mitochondrial oxidoreductases, both chemical compounds were measured colorimetrically using a CLARIOstar microplate reader (BMG-Labtech, Country). Results were expressed as percentage cell viability compared with control cells. The cytotoxicity assay was carried out on two independent days.

### Statistical Analysis

Statistical comparisons were made on the basis of an experimental design with a 95% confidence level, and the results were expressed as the mean ± the standard deviation. Data analysis was performed by using Statgraphics Centurion 16.103 software. Different letters indicate significant differences in parameters between samples. A p-value of less than 0.05 indicates that the means between samples are significantly different.

## Results and Discussion

### Structural Characterization by FTIR and GPC

The FTIR spectra of the films with a plasticizer (Plast) and a CE are shown in Figures 1 and 2, respectively. As it was expected, all FTIR spectra displayed the characteristic peaks of the PLA polymer. The peaks at around 3000 cm^–1^ corresponded to the stretching vibrations of the C–H bond; the strong absorption peak at 1750 cm^–1^ was associated with the stretching vibrations of the carbonyl group (C=O), while the peak at 1453 cm^–1^ corresponded to the CH_3_ group.^25^ The peaks at 1388 cm^–1^ and 1366 cm^–1^ were assigned to symmetric and asymmetric deformation of C–H, respectively, the peak at 1264 cm^–1^ was related to C=O bending, and the peaks in the region of 1182 cm^–1^ to 1085 cm^–1^ corresponded to C–O stretching modes. Finally, the peaks at 1044 cm^–1^, 956 cm^–1^, and 868 cm^–1^ were assigned to OH bending, CH_3_ rocking modes, and C–C stretching, respectively.^13,26^

**Figure 2:**
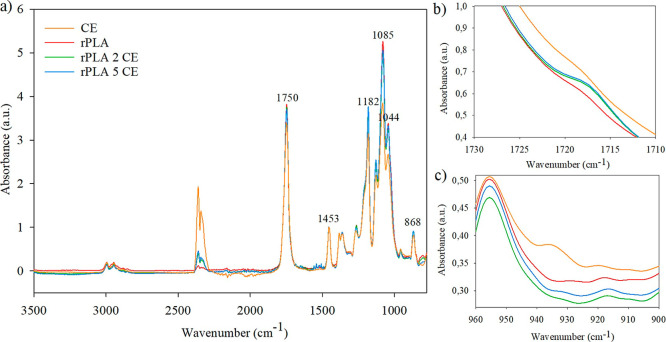
Comparison of the FTIR spectra of CE, rPLA, and films with different CE concentrations between: (a) 850 and 3500 cm^–1^; (b) 1710 and 1730 cm^–1^; and (c) 900 and 960 cm^–1^.

The FTIR spectra of the films with additives showed similar molecular structures, with the same characteristic peaks of rPLA. However, Figures 1b and 2b show that a slight shoulder appeared at approximately 1717 cm^–1^ with the addition of both additives, which is related to a shift in the C=O stretching band that is specific to PLA.^27^

On the other hand, the bands at 922 cm^–1^ and 912 cm^–1^ are related to the crystalline phase of PLA and α and β-crystals, respectively, while the band at 955 cm^–1^ is related to the amorphous fraction.

As can be seen in Figures 1c and 2c, the latter was present in all samples, while those related to crystals were predominant in samples containing additives (especially that of β-crystals), indicating that these samples probably presented a higher crystallinity degree.^28,29^

The molecular weight of the samples was tested using GPC, and the results are shown in Table 1. The results for the rPLA samples with and without additives were compared with the original PLA-based bottle (Bot) and the bottle after the recycling process simulation (Post-consumer PLA, PC-PLA). The results showed the impact of the recycling process simulation and subsequent reprocessing. It was clear how recycling and reprocessing exhibited a great effect on reducing the molecular weight in number ( ${\mathrm{M̅}}_{n}$ ) and in weight $\left({\mathrm{M̅}}_{w}\right)$ of PLA.

**Table 1:** GPC Results of the Developed Composites Compared with the Original PLA-Based Bottle and the Post-Consumer Bottle after the Recycling Process Simulation (PC-PLA)^a^

sample	${\mathrm{M̅}}_{n}$ (g/mol)		${\mathrm{M̅}}_{w}$ (g/mol)	
Bot (PLA)	76880 ± 2946^g^		155396 ± 3836^f^	
PC-PLA	63932 ± 1584^f^		131326 ± 2940^e^	
rPLA	55273 ± 3064^e^		119500 ± 9212^d^	
rPLA 20 Plast	37065 ± 721^b^	507 ± 30^a^	78638 ± 486^b^	796 ± 38^a^
rPLA 30 Plast	26341 ± 308^a^	509 ± 4^a^	51933 ± 419^a^	825 ± 8^a^
rPLA 2 CE	49005 ± 346^d^		101161 ± 1408^c^	
rPLA 5 CE	45010 ± 431^c^		100228 ± 1642^c^	

a The lowercase letters a–g indicate significant differences between the values of the samples for the same parameter according to the ANOVA analysis and Fisher’s LSD test (*p* < 0.05).

Specifically, the original molecular weight of the PLA bottle decreased by 15% in terms of ${\mathrm{M̅}}_{w}$ after the recycling simulation (PC-PLA), whose reduction increased up to 23% after the reprocessing (rPLA) probably due to the combined effect of temperature and mechanical shear during extrusion, as previously reported by other authors.^30^ During mechanical recycling, PLA is susceptible to hydrolytic degradation, which involves the scission of ester bonds, resulting in chains with hydroxyl and carboxyl end-groups. This alters the molar mass distribution of the polymer, directly affecting its physicochemical and mechanical properties.^31^

A reduction in molecular weight was also observed when additives were introduced; this was particularly evident with plasticizer addition, reaching reductions of approximately 34% and 57% when 20 and 30 wt % plasticizers were used, respectively. Similar results were observed by Avolio et al. (2015) that reported the impact of two OLAs (OLA-COOH and OLA-OH) on the physical, thermal, and mechanical properties of PLA. They also confirmed that by adding both oligomers, the molecular weight of PLA decreased,^30^ evidencing that the chain scission increased as plasticizer content increased. In this work, a similar effect was observed; the presence of the OLA-based functional groups, common to both additives, contributed to chain scission during reprocessing, which could partially counteract the chain-extension reactions. Thus, in the case of CE addition, the values of the molecular weight of the resulting composites also decreased with respect to the rPLA control sample. When compared to composites containing plasticizers, an increase in molecular weight was evidenced, since CE is known to react with the carboxyl or hydroxyl end-groups of PLA chains, thereby increasing their molecular weight^32^ and reattaching degraded chains.^33^

At the same time, while all samples showed a single molecular weight result, those containing the plasticizer also exhibited a low-intensity secondary peak with an ${\mathrm{M̅}}_{w}$ of approximately 800 g/mol. The presence of this secondary peak may be related to the acid lactic oligomers of this plasticizer; the addition of higher concentrations of this additive led to excessive interaction with the PLA polymer chains and was found in free form.^34^

### Thermal Characterization

The films were characterized by DSC to check the effect of the addition of the plasticizer and the CE on the thermal parameters of the post-consumer PLA polymer. Figure 3 shows the DSC thermograms, and Table 2 summarizes the main thermal parameters obtained during the first and second heating processes. Depending on the parameter to be analyzed, the most suitable thermogram was selected. As expected, it was observed that the addition of the additives caused significant changes in their thermal properties.

**Figure 3:**
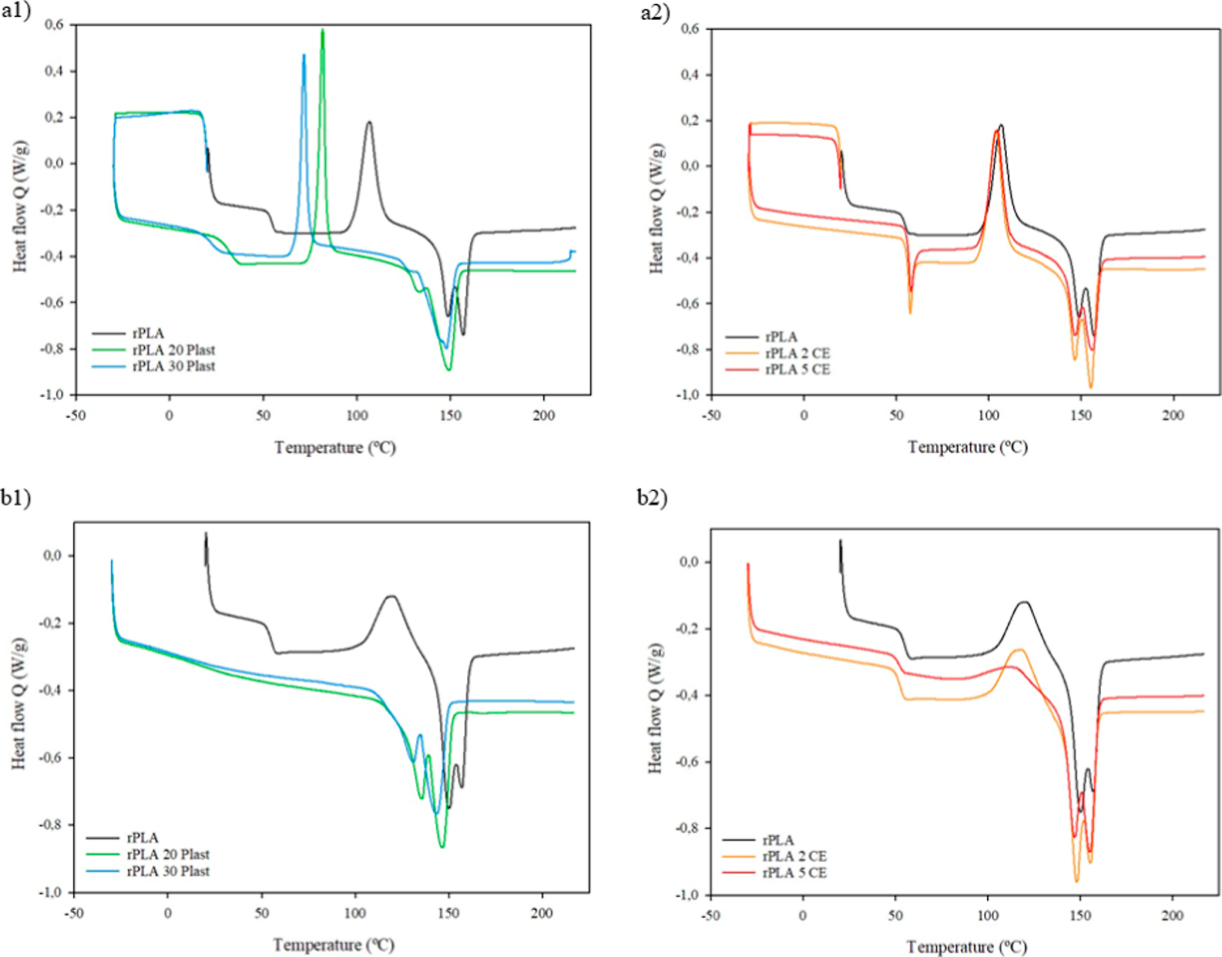
DSC thermograms of rPLA control and rPLA composites with different concentrations of additives during (a) first heating and (b) second heating processes (1—plasticizer; 2—CE).

**Table 2:** Thermal Properties by DSC and Maximum Degradation Temperature (*T*_deg_) by TGA Analysis^a^

sample	heating	*T*_g_ (°C)	*T*_cc_ (°C)	Δ*H*_cc_ (J/g)	*T*_m1_ (°C)	*T*_m2_ (°C)	Δ*H*_m_ (J/g)*	χ_c_ (%)	*T*_deg_ (°C)
rPLA	first	54.1 ± 0.6^e^	106.9 ± 0.6^d^	30.0 ± 1.6^d^	148.5 ± 0.2^de^	156.3 ± 0.9^f^	32.0 ± 0.0^a^	2.2 ± 1.7^a^	317.6 ± 7.7^a^
	second	54.8 ± 0.1^gh^	118.4 ± 1.5^g^	20.7 ± 0.2^c^	149.7 ± 0.1^e^	157.0 ± 0.2^g^	35.7 ± 1.1^d^	16.2 ± 1.0^d^	
rPLA 20 Plast	first	31.7 ± 0.4^b^	82.1 ± 0.5^b^	28.6 ± 2.3^d^	137.7 ± 2.0^b^	148.8 ± 0.7^c^	34.3 ± 0.2^bc^	6.2 ± 2.3^b^	325.5 ± 29.2^ab^
	second				137.4 ± 3.5^b^	145.8 ± 0.2^b^	43.0 ± 1.0^h^	46.3 ± 1.1^g^	
rPLA 30 Plast	first	21.9 ± 0.5^a^	72.0 ± 0.2^a^	22.2 ± 0.3^c^	147.5 ± 0.4^cd^		33.2 ± 0.2^b^	11.8 ± 0.5^c^	340.0 ± 15.2^b^
	second				131.3 ± 0.8^a^	143.1 ± 0.6^a^	39.8 ± 0.5^f^	42.8 ± 0.5^g^	
rPLA 2 CE	first	54.7 ± 0.2^f^	104.0 ± 0.4^c^	35.0 ± 0.6^e^	146.5 ± 0.4^c^	155.0 ± 0.4^de^	35.3 ± 1.1^cd^	0.4 ± 0.5^a^	370.0 ± 1.5^c^
	second	51.9 ± 0.1^d^	116.5 ± 2.0^f^	17.2 ± 1.4^b^	147.8 ± 0.2^cd^	155.5 ± 0.1^e^	40.1 ± 1.2^f^	24.7 ± 2.8^e^	
rPLA 5 CE	first	55.2 ± 0.5^h^	103.9 ± 0.4^c^	28.8 ± 1.8^d^	147.1 ± 0.8^cd^	155.5 ± 0.1^e^	37.6 ± 1.8^e^	9.5 ± 3.8^c^	366.9 ± 5.6^c^
	second	51.5 ± 0.1^c^	113.3 ± 0.7^e^	12.4 ± 0.4^a^	146.9 ± 0.4^cd^	154.8 ± 0.2^d^	41.6 ± 0.7^g^	31.3 ± 0.3^f^	

a The lowercase letters a–h indicate significant differences between the values of the samples for the same parameter according to the ANOVA analysis and Fisher’s LSD test (*p* < 0.05). *The melting enthalpy is the sum of both melting peaks.

During the first heating, rPLA presented a *T*_g_ of around 54 °C, in accordance with our previous work.^35^ A significant decrease in the glass transition temperatures was observed with the addition of the plasticizer, reducing the *T*_g_ values to approximately 31.7 and 21.9 °C when the plasticizer was added at 20 and 30 wt %, respectively. According to the results shown in Table 2, the presence of the plasticizer significantly increased the mobility of the PLA molecular chains, which in turn affected the crystallization process.^36^ This resulted in an increase in the crystallinity of these films during the first heating. In contrast, the addition of CE did not show a clear trend during the first heating. Still, in the second heating when the thermal history was erased, crystallinity values increased with the addition of both the plasticizer and CE, without a significant difference between 20 and 30 wt % plasticizers. In addition, it can be seen in the first heating that the temperature of cold crystallization (*T*_cc_) associated with the rearrangement of the amorphous regions decreased drastically with increasing plasticizer content, as PLA crystallization occurred at lower temperatures due to improved chain mobility. This behavior is also reflected in Figure 3a, where the exotherm associated with cold crystallization is more pronounced in films with the plasticizer compared to those with the CE, which is characteristic of plasticized thermoplastics due to this improved chain mobility.^37^

Regarding the melting transition, *T*_m_ showed double melting peaks (*T*_m1_ and *T*_m2_) in the samples.^38^ The rPLA 30 Plast sample showed a slight shoulder in the melting transition around 135 °C and a clear peak at 147.5 °C (Figure 3a). During the second heating (Figure 3b), it was clearly observed that the melting temperatures decreased with the addition and concentration of the plasticizer and CE, but this difference was more significant with the addition of the plasticizer (Table 2). Nevertheless, the melting processes occurred in a similar temperature range for all samples.

Interestingly, as Figure 3b1 shows, the glass transitions of the samples with the plasticizer disappeared during the second heating process, which evidenced a highly ordered structure, and therefore, a cold crystallization process was not observed for these samples. On the other hand, the *T*_g_ of composites containing the CE was presented as an endothermic transition characteristic of molecular relaxation during the first heating (Figure 3a2) that disappeared during the second heating (Figure 3b2). Thus, the glass transition of these composites was evaluated after the thermal history was erased during the second heating process, and thermograms in Figure 3b displayed that the *T*_g_ values shifted slightly a few degrees lower, as shown in Table 2, accompanied by an increase in crystallinity degree. Furthermore, the rPLA and CE-containing composites showed a slight increase in *T*_cc_ during the second heating process, while the melting processes remained almost constant, as can be seen in Table 2.

TGA analyses were also carried out to determine the degradation temperature (*T*_deg_) of the composites and the thermal stability of the additives as they were melt-blended with the polymer at high temperatures. Figure 4 shows the TGA and derivative (DTGA) curves for each composite, and Table 2 shows the temperature of maximum degradation, *T*_deg_.

**Figure 4:**
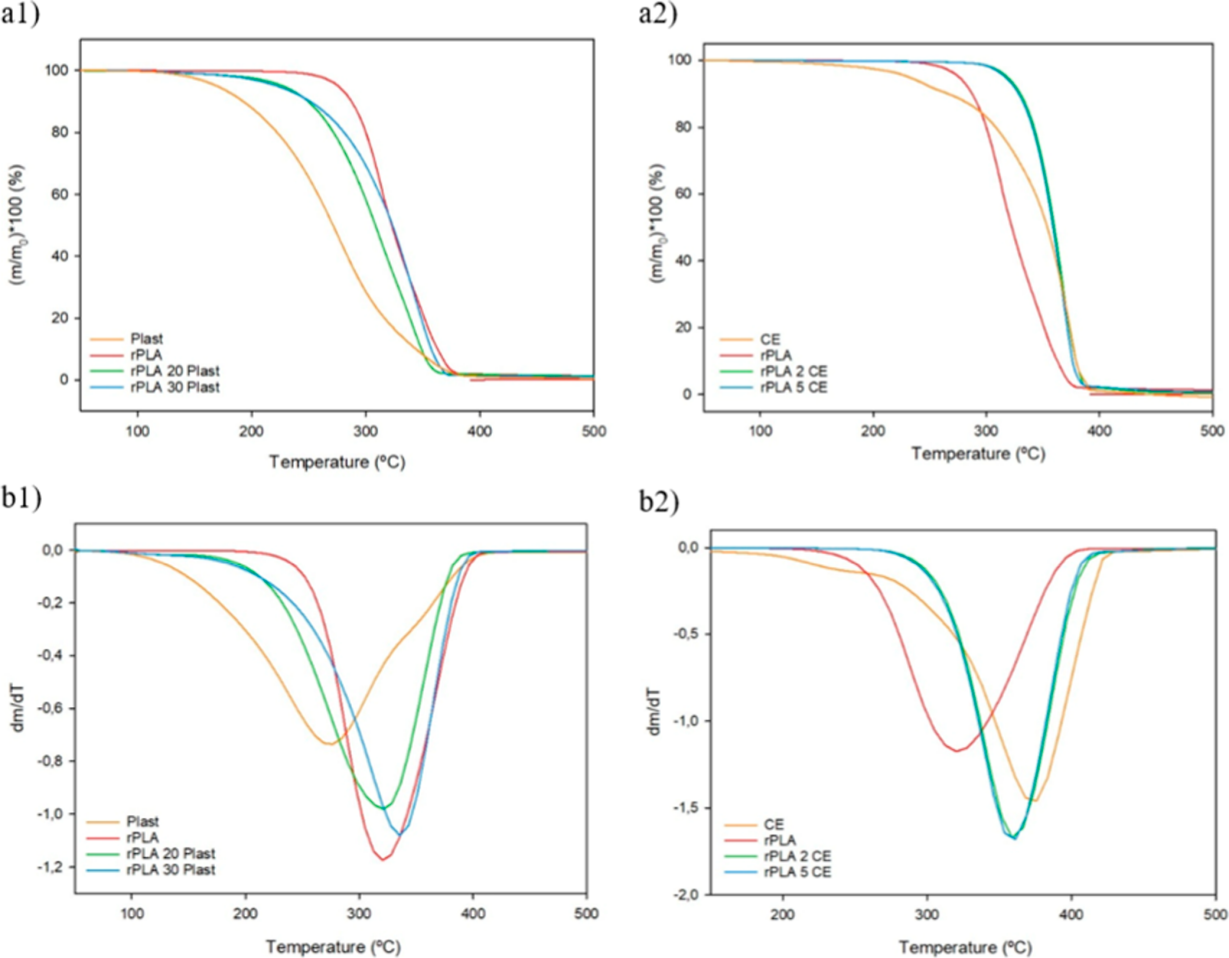
(a) TGA and (b) DTGA thermograms of both additives, rPLA, and films with different additive concentrations (1—plasticizer; 2—CE).

The *T*_deg_ of the plasticizer-containing samples was lower than that of the CE-containing samples because the *T*_deg_ of the plasticizer was much lower than that of the CE (282 °C vs 375 °C). Nevertheless, the stability of rPLA was improved by the addition of both additives, since their degradation occurred at higher temperatures.

### Water Vapor Permeability and Mechanical Characterization

Table 3 shows the results of the WVP of rPLA control and developed rPLA composites at different RH. At RH 50 and 90%, the composite with a low plasticizer concentration exhibited the lowest WVP values, decreasing around 13.5 and 4%, respectively, with respect to control rPLA. However, when the plasticizer concentration increased up to 30 wt %, a significant 28% increase in the WVP value at RH 90% was observed, while the increase of WVP at RH 50% was not significantly different with respect to control rPLA. Previous studies have already reported that, at low concentrations, plasticizers can decrease the WVP of PLA films, but at higher concentrations, WVP often increases. Higher plasticizer levels disrupt the polymer matrix, increase free volume, and enhance chain mobility, making it easier for water vapor to permeate. Lim et al. (2015) reported that the WVP of the PLA composites decreased by around 24% with respect to PLA control with the addition of 3 wt % acetyl tributyl citrate (ATBC), as a plasticizer, while WVP increased significantly to higher values when added at 4 wt %.^39^

**Table 3:** Water Vapor Permeability (WVP) of the Films at Both Relative Humidities^a^

WVP × 10^15^ (kg m/m^2^ s Pa)		
sample	RH 50%	RH 90%
rPLA	6.98 ± 0.36^b,*x*^	10.41 ± 0.73^ab,*y*^
rPLA 20 Plast	6.04 ± 0.33^a,*x*^	9.99 ± 0.34^a,*y*^
rPLA 30 Plast	7.26 ± 0.40^b,*x*^	13.36 ± 0.62^c,*y*^
rPLA 2 CE	6.67 ± 0.66^ab,*x*^	11.01 ± 0.23^ab,*y*^
rPLA 5 CE	6.59 ± 0.14^ab,*x*^	11.36 ± 0.18^b,*y*^

a Lowercase letters a–c indicate significant differences in WVP between samples at the same RH; and lowercase letters *x* and *y* indicate significant differences between the RH of the same sample.

At the same time, when CE was added at both concentrations, the water vapor barrier of the resulting composites was improved, although the rate of improvement was relatively low, approximately 4.5% and 1.1% for composites with the CE at 2 and 5 wt %, respectively. This improvement could probably be increased by increasing the CE concentration. It has been previously reported that one of the main factors influencing permeability is crystallinity, as polymers with high crystallinity are less permeable due to their ordered structure, which reduced free volume, and, in turn, reduced their permeability.^40^ As reported in Table 1, composites with both additives exhibited higher crystallinity values than control rPLA, which can explain their improvement in water vapor barrier.

All samples exhibited an increase in WVP as the RH increased. In this context, the service life and recycling processes could have degraded and increased the material’s sensitivity to humidity, since it can cause structural changes, such as a reduction in molecular weight or an increase in the amorphous fraction. The chain scission results in the increase of hydrophilic end-groups and thus greater sensitivity to humidity. When compared to control rPLA, only the composite containing 20 wt % plasticizer improved the water vapor barrier by about 4%, while the addition of the highest plasticizer concentration caused an even greater increase in WVP value. This fact is in agreement with GPC values that confirmed that the excessive presence of this additive increased the chain scission and, therefore, the hydrophilicity of this composite. At the same time, the addition of 2 and 5 wt % CE led to an increase in WVP values but not as noticeable as the addition of 30 wt % plasticizer. The addition of both additives probably increased the hydrophilic groups in the samples derived from their oligomeric-based structures, and therefore, the increase of RH induced a greater increase in WVP values.

Considering these results, the WVP values of the developed composites did not differ significantly from control rPLA, particularly at low RH. At RH 90%, however, the results depended on the type of additive and the structural properties. Overall, these films could be used in applications where a water vapor barrier is not a major limitation.

The tensile properties of the control and rPLA composites are presented in Table 4. As expected, the addition of the plasticizer resulted in a statistically significant reduction in stiffness at 30 wt % Plast and in the maximum strength. Stiffness is measured by YM, which is calculated as the slope of the linear region of the stress–strain curve. The ductility of rPLA, i.e., its ability to elongate, was drastically enhanced by the addition of a 30 wt % plasticizer. This property was modified by a factor of 26 compared to rPLA. Plast molecules were located between the PLA chains, reducing rPLA chain entanglements and increasing the polymer’s mobility during traction. However, the reduced interactions between the PLA chains also decreased their resistance. Interestingly, the addition of 20 wt % plasticizer did not affect the ductility and stiffness of this rPLA. This same trend has been observed by Delicado et al. (2022), who reported a slight reduction in the ductility, a nonsignificant change in YM, and a marked decrease in the TS of extruded-injected virgin PLA (99% l-isomer) with 10% Glyplast OLA 2.^41^ This indicated that there is a critical concentration of Glyplast OLA 2 (more than 20 wt %) above which the molecules of this plasticizer, a low-molecular-weight oligomeric lactic acid, begin to disperse uniformly among PLA chains, plasticizing it rather than causing tension points. Below this amount, the plasticizer molecules are unable to separate the PLA chains fully and the plastic does not achieve the desired ductility.

**Table 4:** Mechanical Parameters of the Films Obtained through Tensile Tests^a^

sample	tensile strength (MPa)	Young’s modulus (MPa)	elongation at break (%)
rPLA	63.4 ± 2.8^c^	2468 ± 542^b^	48.7 ± 4.2^a^
rPLA 20 Plast	48.1 ± 2.2^b^	2494 ± 410^b^	31.8 ± 3.8^a^
rPLA 30 Plast	24.7 ± 8.0^a^	1237 ± 314^a^	1287.7 ± 115.6^b^
rPLA 2 CE	64.0 ± 19.0^c^	2423 ± 72^b^	40.1 ± 5.2^a^
rPLA 5 CE	64.4 ± 3.1^c^	2200 ± 179^b^	41.1 ± 2.3^a^

a The lowercase letters a–c indicate significant differences between the values of the samples for the same parameter according to the ANOVA analysis and Fisher’s LSD test (*p* < 0.05).

The incorporation of 30 wt % plasticizer increased the elongation at break to 1287% (see Figure SM2 of the Supporting Information). This substantial improvement is attributed to the pronounced decrease in *T*_g_ (from 54.1 to 21.9 °C) and the enhanced chain mobility imparted by the plasticizer, which facilitated extensive plastic deformation. Nevertheless, such high plasticizer loadings may induce phase separation, morphological changes, and reduced mechanical stability due to potential plasticizer migration. Therefore, despite the remarkable increase in ductility, the long-term suitability and effectiveness of this plasticizer in rPLA should be critically evaluated. Comparable high elongation values have been previously reported for PLA materials processed under specific conditions or blended with appropriate modifiers. For instance, PLA blended with polyethylene glycol (20 wt %) and maleic anhydride-grafted PLA (5 wt %) via melt mixing and molding reached an elongation at break of 526%, whereas self-nanofibrillated PLA displayed excellent mechanical performance with well-balanced strength, ductility, and toughness, achieving approximately 329% elongation at break.^42,43^

Although the crystallinity of rPLA increased with the addition of a plasticizer due to the promotion of the spherulitic growth reported in the literature for virgin PLA,^44^ the stiffness and TS of rPLA decreased. This suggests that the reduction in chain entanglements caused by the plasticizer had a greater effect on the mechanical parameters than the chain ordering. Chieng et al. (2013) observed a similar trend, indicating an inverse relationship between TS and modulus as crystallinity increased. However, an increase in elongation at break when poly­(ethylene glycol) was added to virgin PLA was also reported.^45^

Conversely, the tensile properties of rPLA remained unaffected by the addition of CE at both concentrations, as demonstrated by the statistical analysis in Table 4. The crystallinity of rPLA increased with the addition of CE at 5 wt % (9.5%, as Table 2 shows), suggesting that this concentration of CE molecules facilitated the formation of longer PLA chains that, in turn, led to increased chain folding. However, this chain extension did not affect the tensile parameters. Previous results reported that the addition of the Joncryl CE to two-times reprocessed PLA in a lab-scale simulated recycling process exhibited an 8-fold increase in crystallinity without significantly affecting tensile properties.^46^

### Global Migration

Global migration studies have been carried out in terms of food safety, as migration of substances from plastic packaging can be a risk in addition to noncompliance. The aim was to test the total amount of nonvolatile compounds that could be transferred from the developed composites to the food in case these composites are applied as food contact materials. The results of the global migration are presented in Table 5. Because according to Article 12 of European Regulation 10/2011, the global migration limit is set at 10 mg/dm^2^, it was evidenced that composites with both plasticizer concentrations exceeded the limit values. However, the values obtained in aqueous Sim A and water tended to be significantly lower than those in the fatty food simulant (Sim D1), possibly due to the low water solubility of the plasticizer. Practically more than half of the plasticizer content of both composites migrated into Sim D (approximately 13% and 19% for composites with 20 and 30 wt % plasticizer, respectively), while only 5% and 6% of the plasticizer migrated in Sim A and 3% and 7% in water, indicating that the plasticizer really had a good compatibility with rPLA. At the same time, the temperature during the contact period was undoubtedly an important factor that influenced these values. Considering that the *T*_g_ of the samples was quite lower than 40 °C, phase separation and, therefore, increased migration of components occurred. This hypothesis was supported by the results obtained at 10 °C, where migration levels were significantly lower than those obtained at 40 °C. Similar results were reported by Mutsuga et al. (2008) that performed migration studies of lactic acid, lactide, and oligomers at temperatures above and below the PLA *T*_g_ and at different contact times. In general, the authors concluded that global migration was higher at temperatures above *T*_g_ than that at lower temperatures, even for shorter contact times.^47^ In this case, the percentage of the plasticizer that migrated was considerably lower than at 40 °C: 1 and 3% for 20 and 30 wt % plasticizer, respectively, in Sim A and water, and 1.4 and 6% for 20 and 30 wt % plasticizer, respectively, in Sim D. This further supports the theory that the temperature affected global migration processes.

**Table 5:** Global Migration (GM) Values of the Films in Water and Food Simulants at 10 and 40 °C^a^

*T* (°C)	sample	sim A (mg/dm^2^)	sim D1 (mg/dm^2^)	water (mg/dm^2^)
10 °C	rPLA	0 ± 0^a^	0 ± 0^a^	0 ± 0^a^
	rPLA 20 Plast	4.83 ± 2.59^b,*x*,1^	10.00 ± 0.47^b,*z*,1^	7.00 ± 0.00^c,*y*,1^
	rPLA 30 Plast	13.83 ± 2.12^c,*x*,1^	25.17 ± 5.42^c,*y*,1^	12.67 ± 0.94^d,*x*,1^
	rPLA 2 CE	0.67 ± 0.94^a,*x*,1^	0.50 ± 0.24^a,*x*,1^	0.83 ± 0.24^b,*x*,1^
	rPLA 5 CE	0.67 ± 0.47^a,*x*,1^	1.17 ± 0.24^a,*y*,1^	0.50 ± 0.24^a,b,*x*,1^
40 °C	rPLA	0 ± 0^a^	0 ± 0^a^	0 ± 0^a^
	rPLA 20 Plast	15.33 ± 5.19^b,*x*,2^	54.17 ± 2.12^b,*y*,2^	22.00 ± 8.01^b,*x*,2^
	rPLA 30 Plast	22.67 ± 0.47^c,*x*,2^	55.83 ± 3.54^b,*z*,2^	30.67 ± 3.30^c,*y*,2^
	rPLA 2 CE	0.83 ± 1.18^a,*x*,1^	0.83 ± 0.24^a,*x*,1^	1.17 ± 0.71^a,*x*,1^
	rPLA 5 CE	0.50 ± 0.24^a,*x*,1^	1.67 ± 0.47^a,*y*,1^	0.50 ± 0.71^a,*x*,1^

a Lowercase letters a–c indicate significant differences between samples in the same simulant at the same temperature; lowercase letters *x*–*z* indicate significant differences between simulants of the same sample at the same temperature; and numbers 1 and 2 indicate significant differences of the same sample at different temperatures in the same simulant.

For samples containing the CE, the global migration values obtained were well below the established limit, which can be translated into a high chemical affinity between the polymer and the CE, and this additive probably reacted with the rPLA terminal of groups, as expected. In addition, in these composites, the thermal exposure did not affect the global migration, probably because their *T*_g_ was higher than both experimental temperatures. It is interesting to point out that the global migration of rPLA was null in all cases.

### Effect of OLA-Based Additives on Intestinal Cells

Aqueous migration solutions of rPLA as well as Plast and CE-containing rPLA composites were evaluated on Caco-2 cells since the intestinal epithelium was exposed to water and food constituents after ingestion. The results of the cell viability study after 24 h of exposure are shown in Figure 5. The viability percentage (%) data were 102.6 ± 3.6 (rPLA), 95.7 ± 8.2 (rPLA 20 Plast), 70.2 ± 7.9 (rPLA 30 Plast), 103 ± 2.2 (rPLA 2 CE), and 95.2 ± 2.9 (rPLA 5 CE) compared to control cells (99.6 ± 6.4). Because cell viability is considered to be affected when values are below 80% viable cells, only exposure to migrating substances from the composite developed with 30 wt % plasticizer reduced the mitochondrial activity of intestinal cells, achieving 70% cell viability compared to the control (100% cell viability). All other samples, including control post-consumer rPLA biodegradable plastic, have shown null toxicity. These data are consistent with the migration values of additives reported in water at 40 °C (Table 5), as a greater additive migration leads to the onset of intestinal toxicity.

**Figure 5:**
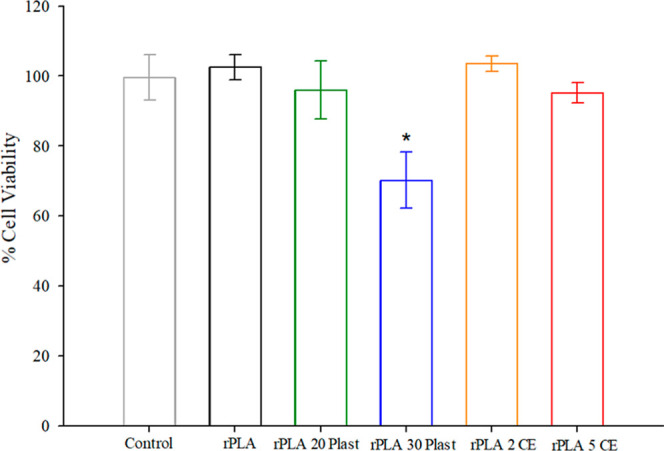
Cell viability of Caco-2 monolayers after exposure to aqueous migration solutions from control rPLA and rPLA composites for 24 h. Results expressed as percentage cell viability with respect to control cells (mean ± SD, *n* = 6). The asterisk (*) indicates a statistically significant decrease (*p* < 0.05) in mitochondrial activity compared to control cells.

In a recent study, a branched lactic acid ester plasticizer for poly­(vinyl chloride) (PCV) materials was synthesized, and its application performance and oral toxicity were investigated.^48^ The findings showed that the novel lactic-acid-derived plasticizer improved the properties of the plastic material and did not cause toxic effects or mortality in rats after oral administration. The authors then proposed this nontoxic plasticizer as a sustainable option to replace petroleum-based plasticizers.

In this context, plasticizers were the most abundant additives found in PLA and poly­(hydroxybutyrate) (PHB) materials.^49^ Cytotoxicity assays using A549 human lung and HepG2 human liver cancer cell lines showed that additives did not reduce cell viability but did generate oxidative stress. The overall conclusion of this study was that biobased materials can represent similar risk for the environment and human health as fossil-based materials if they include toxic additives. This situation highlights the need to search for additives, particularly plasticizers derived from renewable sources with proven nontoxic properties.

## Conclusions

Composites of post-consumer rPLA derived from commercial water bottles were developed by incorporating OLA-based additives, namely, a plasticizer and a CE. Adding these additives to rPLA significantly altered its final properties. First, the addition of the OLA-based functional groups, common to both additives, contributed to chain scission during reprocessing, which could partially be counteracted by chain-extension reactions. A more significant reduction in the molecular weight was observed when the plasticizer was used. Although the concentration of the CE used in this study was relatively low, increasing its amount would likely not lead to further improvements, as the observed degradation phenomenon would limit any potential molecular weight increase. Therefore, achieving a more pronounced effect may require the use of an alternative CE. Meanwhile, the thermal stability of rPLA composites improved, shifting their degradation toward higher temperatures. Conversely, the addition of the plasticizer reduced the glass transition temperature and improved flexibility but at the cost of lower mechanical strength and higher migration. The use of the CE allowed for better mechanical strength and migration values that were well below the established limit. Overall, the WVP was not significantly affected by the incorporation of the additives, with the exception of the plasticizer at low concentrations, which improved the barrier properties, likely due to enhanced structural cohesion. Ultimately, choosing the right additive will be key to optimizing the performance of rPLA. Depending on the application and the required final properties of the material, a plasticizer or CE can be chosen.

The combined approach here proposed, linking migration data with cell viability in an intestinal cell model, contributes to a more robust food safety assessment for the potential reuse of the biobased material under study. Overall, these findings demonstrate a practical route toward the sustainable reuse of PLA, showing that combining mechanical recycling with the use of additives can optimize the material’s functionality without compromising safety. This supports its incorporation into food packaging applications within a circular economy model.

## Supplementary Material



## Notes

Description of PLA-based water bottles: Figure S1 presents a photograph of commercial PLA-based water bottles; and Figure S2 presents a picture that describes the mechanical tests of the plasticized rPLA 30 Plast sample (PDF)

## Abbreviations

ATCC: American type culture collection
CE: chain extender
DMEM: Dulbecco’s Modified Eagle’s Medium
DSC: differential scanning calorimetry
EDTA: ethylenediaminetetraacetic acid
FBS: fetal bovine serum
FTIR: Fourier transform infrared
GPC: gel permeation chromatography
OLAs: acid lactic oligomers
PC-PLA: post-consumer recycled bottle
PCV: poly­(vinyl chloride)
PHB: poly­(hydroxybutyrate)
PLA: polylactic acid
PDLA: poly­(d-lactic acid)
PLLA: poly­(l-lactic acid)
RH: relative humidity
TGA: thermogravimetric analysis
WVP: water vapor permeability
