# Supplementary material for: Sustainable Upgrade of Post-Consumer PLA: The Effect of Adding a Plasticizer and a Chain Extender on the Functional Properties and Toxicity of This Recycled Bioplastic
Source: ACS Omega. 2025 Dec 22;11(1):1690–702. doi: 10.1021/acsomega.5c09603 (PMC12809588; doi:10.1021/acsomega.5c09603)
Supplement: Supplementary file 1 [file ao5c09603_si_001.pdf]

Sustainable Upgrade of Post-Consumer PLA: Effect of Adding a Plasticizer and a Chain Extender on the Functional Properties and Toxicity of this Recycled Bioplastic

Diana Morán [a], *Eliezer Velásquez* [b,c], *Marta Arroyo Calatayud* [d], *Beatriz de la Fuente* [d], *Pilar Hernández-Muñoz* [a], *Carol López-de-Dicastillo* [a*]

[a] Packaging Group, Institute of Agrochemistry and Food Technology (IATA-CSIC), Av. Agustín Escardino 7, 46980 Paterna, Valencia, Spain.

[b] Packaging Group Innovation Center (LABEN-Chile), University of Santiago of Chile (USACH), Santiago 9170201, Chile.

[c] Center for the Development of Nanoscience and Nanotechnology (CEDENNA), University of Santiago of Chile (USACH), Santiago 9170124, Chile

[d] Research Group in Integrative Approaches in Food Toxicology, Institute of Agrochemistry and Food Technology (IATA-CSIC), Av. Agustín Escardino 7, 46980 Paterna, Valencia, Spain.

Materials

Figure SM1 shows a photograph of commercial green polylactic acid (PLA)-based water bottles.

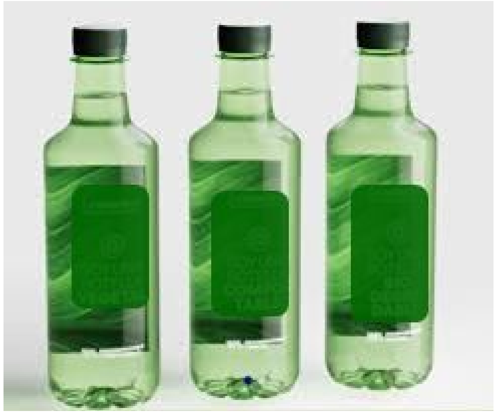

Figure S1. PLA-based water bottles.

Mechanical tests

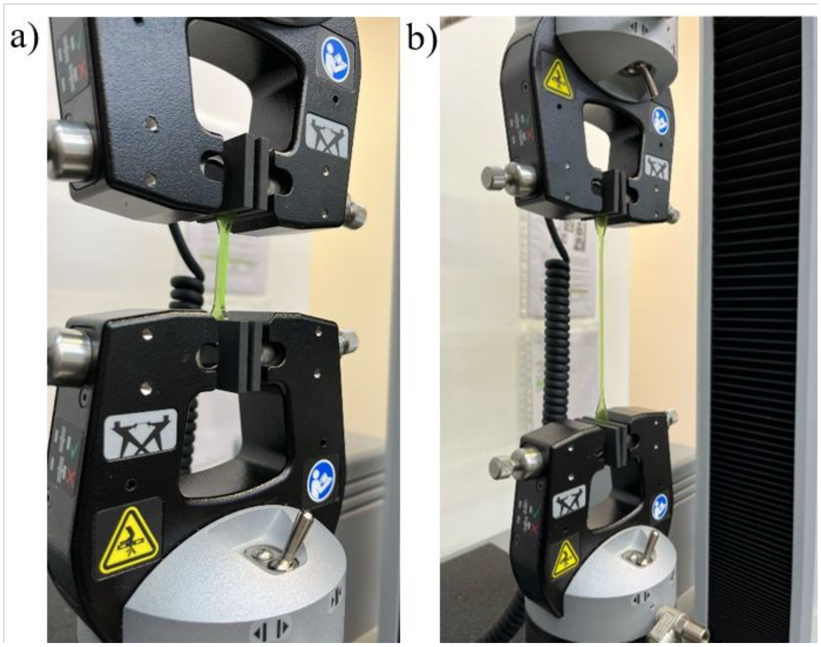

Figure S2. Mechanical test of rPLA 30Plast: a) in the beginning of the test; and b) during the test.
